# Association Between Climate Change Awareness and Depression & Anxiety: Findings from a U.S. Sample

**DOI:** 10.3390/ijerph22091426

**Published:** 2025-09-12

**Authors:** Stanley Nkemjika, Colvette Brown, Henry Onyeaka, Srikanta Banerjee, Jennifer A. Oliphant

**Affiliations:** 1 Department of Psychiatry and Human Behavior, Thomas Jefferson University, Philadelphia, PA 19107, USA; 2 College of Health and Health Sciences, Walden University, Minneapolis, MN 55401, USA; srikanta.banerjee2@mail.waldenu.edu (S.B.);; 3 Department of Population Health, School of Public Health, Georgia State University, Atlanta, GA 30303, USA; 4 Department of Psychiatry, Harvard Medical School, Boston, MA 02115, USA

**Keywords:** climate change awareness, depression, anxiety, sociodemographic attributes

## Abstract

Background: Exploring the connection between climate change awareness and overall well-being is crucial, particularly in how it impacts both the environment and mental health. The mental health consequences of climate change and its awareness have not been thoroughly examined, especially in the U.S. In this study, the relationship between awareness of climate change and depression or anxiety among U.S. adults was explored. Similarly, the role of climate change awareness has not been explored in relation to mental health concerns. Methods: Based on the HINTS-6 dataset, a nationally representative, cross-sectional survey conducted by the National Cancer Institute, a variety of statistical methods, including logistic regression models, to examine these relationships were used. This study had a sample size of 6154 participants. A statistically significant level of <0.05 was used. Result: The findings showed that individuals who are aware of climate change have a higher adjusted odds ratio of 1.392 (1.160–1.671) for experiencing depression or anxiety compared to those who are unaware. Additionally, non-heterosexual individuals displayed a significantly higher adjusted odds ratio of 2.691 (CI: 2.125–3.407) for depression or anxiety, underscoring the strong connection between mental health and climate change awareness. Conclusion: This study highlights a notable link between heightened awareness of climate change with depression and anxiety among the U.S. population.

## 1. Introduction

Climate change has been characterized as “potentially the greatest global health threat of the 21st century,” with projected adverse effects on both mental and physical health [1]. Thus far, these predictions have been fulfilled. The impact of climate change on mental health may be direct, as individuals are affected by extreme weather events such as intense heat, floods, wildfires, cyclones, and hurricanes [2]. Moreover, a peer-reviewed framework highlights that climate change also exerts substantial indirect effects as well [3]. A study looked at the mental health impacts of flooding in two areas of India, Chennai and Cuddalore, involving 223 individuals directly affected by the floods [4]. The results revealed that approximately 45% of the participants experienced some form of psychiatric distress. Specifically, 101 of the participants (45.29%) were dealing with depression, 60 people (26.9%) showed symptoms of PTSD, and 48 (27.4%) experienced anxiety. Additionally, 11 individuals (4.9%) reported an increase in substance use following the disaster [4]. This finding is supported by a systematic review and meta-analysis of 25 U.S. studies (total N ≈ 616,657; meta-analysis N = 2012), which showed that exposure to hurricanes and flooding was consistently associated with moderate increases in PTSD (g = 0.44; 95% CI 0.04–0.85) and smaller but significant increases in depression (g = 0.28; 95% CI 0.04–0.53), while anxiety showed no meaningful effect (g = 0.05; 95% CI −0.30–0.19), thus highlighting that such natural disasters have a pronounced impact on PTSD and depression among affected populations, with negligible effects on anxiety [5]. Climate change increases the risks of prolonged droughts, food insecurity, habitat loss, land inundation, and salinization due to rising sea levels, deforestation, and resultant forced migration [6,7,8]. These factors may contribute to an increased incidence of various mental health disorders like depression and anxiety [6,9,10,11,12]. For example, Monsour et al. (2022) focused on the impact of climate change-induced sea level rise (SLR) and tropical cyclone exposure on mental illness symptom prevalence, noted that a significant number of people in South Florida were projected to develop anxiety, major depressive disorder, or PTSD symptoms following tropical cyclone exposure due to climate change [13]. Another study found that higher levels of daylight exposure following climate-change induced different cloud cover were associated with improved sleep quality [14]. Hence, it highlights the role of increased natural daylight in winter as a potential way to reduce the likelihood of experiencing depression symptoms.

Climate change significantly affects various aspects of human life, particularly physical and mental health [15,16,17]. Growing evidence in the literature indicates that behavioral and mental health disorders such as major depressive disorder and bipolar disorder are influenced by environmental and climatic conditions [15]. In a similar vein, extreme weather events like heat waves, coastal erosion, and floods can throw agricultural practices into disarray [18]. This situation creates job insecurity for farmers and workers globally while also deepening mental health struggles, which raises serious public health issues. For example, Abbasi discovered that more cloud cover in spring and less sunlight in winter can worsen depressive symptoms and that rising temperatures are linked to greater dissatisfaction among people with bipolar disorder [9]. Additionally, Prencipe et al. found that a significant proportion of the population study reported experiencing climate distress, with approximately 70% indicating feelings of anxiety, depression, and worry related to climate change [19]. Hence, climate change seems to be an essential factor leading to a decrease or increase in stress and a shift in humans’ behavior.

Evidence in the literature has increasingly described heat exposure as a critical climate-related factor impacting mental health stability [2]. As a public health concern, the increasing temperature of heat exposure concerns following climate change is notably reported to lead to psychiatric morbidity in the form of suicides and severe mental health disorders [2,9]. Despite the growing interest in this area, research on how awareness of climate change and different sociodemographic factors affect mental health is still quite limited. Regarding sociodemographics, studies have suggested that children and adolescents are particularly vulnerable to the psychological impacts of climate change [14,16,20,21]. For example, La Greca et al. reported significant physical complaints among children exposed to extreme weather events linked to stress from evacuation and displacement [20]. Research has also indicated that social influences and emotional responses are vital in climate change activism [11,22]. Similarly, evidence suggests a diminished sense of perceived control among the youths following a pandemic-related event, which was associated with increased mental health symptoms [22]. However, the mechanism of the relationship remains understudied. Other findings in the literature suggest that women are disproportionately affected by climate change due to their caregiving roles, which exacerbates psychological distress, particularly during resource scarcity or threats to their children’s safety- PTSD, anxiety, and depression [21]. Notably, financial instability and inadequate access to mental health resources have been described as a mediator of the public health burden [16,19]. Additionally, Prencipe et al. supported this literature as lower socioeconomic backgrounds reported more significant climate distress, evidenced by more adversely affected mental health [19].

Evidence in the literature showed significant concerns about climate change, coupled with anxiety about the future, leading to emotions like fear, anger, and sadness, collectively known as climate anxiety, which may increase substance use disorder and suicidal behavior [23]. For example, a study of Norwegian adults found that while many expressed worry about climate change, especially women and those with higher education, the overall concern was lower compared to adults in European countries like the UK, Spain, and Italy [14,16,21]. Research also indicates that anxiety about climate change is more prevalent among adolescents and young adults [20,21,23,24]. The growing awareness of the negative impact of climate change on mental health, especially among children and adolescents [14,16,21], has not only been in focus in research but also the media. Other notable demographic attributes were adults living in urban areas expressed more worry about climate change than those living in rural areas. Despite these worries about climate change, Norwegian adults were less worried about climate change than adults in other European countries [21]. Leonhardt et al. also reported that almost 40% of Norwegian adolescents were worried about climate change and that girls, pupils with at least one parent with higher education, and pupils from urban areas were more inclined to worry about the climate [21]. Leonhardt et al. further noted that adolescents who were worried about climate change had more symptoms of depression than those who were not so concerned [21]. The role of geographic disparities in mental health outcomes following climate change awareness has been documented in the literature, as there were higher adverse mental health effects among the population in rural/suburban settings compared to their urban counterparts [25]. Studies have indicated that individuals living in rural and suburban areas experience more significant adverse mental health effects compared to those residing in urban environments. These disparities are multifaceted and can be attributed to several contextual factors, including differences in access to mental health care, social support, and vulnerability to climate-related stressors. Rural populations often face limited access to health care services, including mental health professionals, which can hinder their ability to receive timely and adequate treatment [26].

Awareness of climate change has been increasingly recognized as a psychological stressor capable of affecting mental health through various cognitive and emotional mechanisms. One prominent pathway is the rise in climate-related distress, often referred to as “eco-anxiety”, a chronic fear of environmental doom that arises from perceived threats to the planet and future generations [27]. Individuals with high levels of climate change awareness tend to experience greater psychological burden due to their enhanced understanding of irreversible environmental damage and insufficient global action. This emotional engagement can lead to heightened levels of mental health concerns in the form of anxiety, depressive symptoms, and existential dread, particularly among adolescents and young adults who perceive their futures as compromised [28]. Such awareness is compounded by repeated exposure to climate discourse in media, which can reinforce a sense of helplessness and uncontrollability, both of which are established cognitive precursors to affective disorders. Additionally, climate change awareness can disrupt individuals’ emotional and social relationships with the environment, a phenomenon that contributes to “ecological grief” and “solastalgia”—emotions linked to anticipated or actual environmental loss. These affective states are not limited to those directly experiencing climate-related disasters; they also manifest in populations that internalize the global consequences of environmental degradation through empathetic and moral reasoning [29]. As individuals become more attuned to environmental injustices and intergenerational inequities, they may also experience moral distress and a diminished sense of agency. However, these emotional responses can be modulated by protective factors such as climate engagement, collective action, and social support, which have been shown to buffer the adverse mental health impacts of Climate change awareness [30]. Thus, while awareness can be a risk factor for psychological distress, it also provides an opportunity for building psychological resilience through meaningful action and community involvement. Thus, further research on the relationship between climate change-related studies and mental health is needed.

Similarly, Lykin et al. showed that exposure to bushfires resulted in higher climate change-related distress and concern [23]. Hence, there is higher perceived personal vulnerability to the effects of climate change in those with direct exposure to bushfires. Finally, the Raza et al. showed that the impact of daylight exposure on mental health outcomes varied across individuals, as living with children younger than 12 years of age had lower odds of reporting symptoms of depression when exposed to daylight one or more hours per day [14]. So far, these findings have not been replicated in the literature and among the American population. Given the compounded impact of the COVID-19 pandemic on mental health, particularly among young people, Lawrence et al. identified that concurrent exposure to climate change concerns, heightened awareness of environmental issues, and stressors associated with the COVID-19 pandemic significantly contributed to increased levels of anxiety, depression, and stress in youth [22]. Additionally, the pandemic exacerbated existing worry about climate change, leading to perceived increased overall psychological distress. Similarly, an additive synergistic effect has been reported regarding climate change impacting a pandemic-related condition, and vice versa, as high levels of distress from both crises were associated with more severe mental health [22]. Hence, the study highlights the possible comorbid association between climate change, mental health, and sociodemographic characteristics.

Vulnerable populations, particularly those with lower socioeconomic status, are disproportionately affected by climate-related shocks [31]. Younger populations and lower socioeconomic backgrounds have been associated with higher levels of depression about climate stressors [31]. Hence, these studies highlight the prevalence of depression and anxiety linked to climate impacts, emphasizing the urgent need for targeted mental health interventions and further research into climate change awareness and its effects on mental health. Understanding the mental health implications of escalating climate change is essential for formulating effective strategies to support affected populations. Current literature highlights significant gaps that must be addressed, particularly regarding how climate change awareness influences mental health in the form of depression or anxiety and the specific challenges different demographic groups encounter. The current literature reveals important gaps in understanding how climate change awareness directly influences mental health, particularly in the context of depression or anxiety. While some studies have examined the broad mental health impacts of climate-related stressors, there is limited research on how awareness of climate change specifically contributes to emotional distress, and how these effects vary across different demographic groups. Additionally, there is limited studies conducted using American population. This research gap is particularly evident when considering how different factors such as age, socioeconomic status, and other sociodemographic attributes interact with Climate change awareness to shape mental health outcomes. Hence, the objective of this study is to address these gaps by examining the relationship between climate change awareness and mental health outcomes, specifically depression or anxiety among Americans. Secondly, we aim to examine the role of sociodemographic attributes as confounding variables. We hypothesized that there is an association between climate change awareness and depression or anxiety among adults in America controlling for age, gender, marital status and socioeconomic status.

## 2. Materials and Methods

### 2.1. Study Design

In this Health information national trends survey 6 (HINTS-6) study, we used a quantitative, cross-sectional design and relied on secondary data from the extensive dataset collected by the National Cancer Institute (NCI) [32]. The survey was carefully designed with strong statistical methods and reliable sampling techniques to ensure accuracy. In addition to assessing public access to health information, the HINTS-6 survey included questions focused on mental health, climate change concerns, and broader social factors affecting health. This makes the dataset especially useful for exploring how climate change awareness, mental health, and social conditions are connected, particularly in vulnerable populations. Along with assessing public access to health information, the HINTS-6 survey also included questions about mental health, concerns over climate change, and broader social factors that affect health. This makes the dataset especially valuable for exploring how climate change awareness, mental health, and social conditions are interconnected, particularly among vulnerable groups. The secondary nature of the data means that no new participants were recruited for this specific study, but rather, existing data were analyzed to answer research questions related to and its potential relationship with mental health.

### 2.2. Study Population

The HINTS is a comprehensive, nationally representative survey administered by the NCI since 2003. HINTS gathers data from civilian, noninstitutionalized adults aged 18 or older living in the United States. The survey’s primary aim is to assess the American public’s access to and utilization of health information, with a specific focus on cancer across the continuum of care prevention, early detection, diagnosis, treatment, and survivorship. The most recent round, HINTS-6, conducted from 7 March 2022, to 8 November 2022, included a goal of obtaining 7000 completed questionnaires. The survey’s focus areas are broad, ranging from health literacy and misinformation to social determinants of health and telehealth usage. The HINTS-6 study population was selected using a two-stage sampling design. For the first stage, a stratified sample of residential addresses across the United States was selected. This approach ensured diverse geographic and demographic representation, with an emphasis on capturing data from both urban and rural areas. The second stage involved the random selection of one adult per household using the Next Birthday Method, which is commonly used in household surveys to minimize bias. The sampling strategy for HINTS 6 also included oversampling of high minority populations, both in urban and rural areas, to ensure adequate representation of these groups, which have historically been underrepresented in health research. The inclusion of rural communities was a novel aspect of the 2022 administration, with the aim of increasing rural participation in health data collection and addressing health disparities that may exist between urban and rural populations.

### 2.3. Sample Size & Statistical Power

The sample size for the HINTS-6 is very critical towards ensuring the study’s statistical power and generalizability. The HINTS-6 survey aimed to collect 7000 completed questionnaires, targeting a diverse group of civilians, noninstitutionalized adults across the United States. However, a study sample size of 6154 participants was identified. Adequate representation sample of the population was achieved by using a two-staged stratified sampling survey design. This sample size is considered large enough to detect meaningful differences and relationships in the data, particularly for exploring complex public health issues such as climate change anxiety and mental health. Considering the robust sample size, the HINTS-6 dataset provides a comprehensive insight into the relationship between perceived climate change health risk and mental health outcomes, offering statistical power to detect small to moderate effect sizes in the population. The large sample size for the dataset plays an essential role in detecting even small associations between different factors. In public health research, having enough statistical power is crucial for ensuring reliable and helpful findings [33]. Thus, possible in helping to guide policy and intervention efforts [32]. The HINT-6 broad sample ensures that the study can capture differences across various demographic groups, including gender or urban-rural populations distribution, as well as different racial and ethnic communities. This is particularly important for understanding health disparities and how social factors influence health outcomes. Power analysis for a sample of this size typically suggests a high probability of detecting statistically significant relationships, assuming the effect sizes are moderate or large. The stratified sampling design of HINTS 6 strengthens the study by oversampling certain groups, such as urban minorities and rural populations, which have often been underrepresented in health surveys. This approach is essential for gaining a deeper understanding of the unique health needs and mental health concerns of these communities, especially regarding climate change. As a result, the sample design not only boosts the study’s statistical power but also ensures that the findings are more inclusive and reflective of the wider population.

### 2.4. Data Collection

The HINTS-6 used a mixed-mode data collection strategy, which offered respondents the option to complete the survey online or on paper. This flexibility was designed to increase participation across different populations, including those with limited access to the internet. To further boost response rates, particularly from hard-to-reach groups, HINTS 6 included additional mailings with increased incentives for non-respondents offering $5 to $10. The data collection period ran for several months, from March to November 2022, and participants were incentivized with a $2 prepaid monetary incentive, with additional bonuses for completing the survey online. This method of incentivization and increased outreach was aimed at maximizing the response rate and ensuring a diverse sample of respondents.

### 2.5. Measures

The dataset offers a rich variety of variables, with dependent variables related to mental health, including questions such as “Has a doctor or other health professional ever told you that you had depression or anxiety disorder?”. The response was “Yes” or “No.”

The independent variable of interest for this study is the perception of climate change, specifically, “how much do you think climate change will harm your health?” [34]. The responses were “A lot”, “Some”, “A little”, “Not at all”, “Don’t Know.” These responses will be recategorized as a dichotomous response (a) Yes for “A lot”, “Some”, “A little”; (b) No for “Not at all”. “Don’t know” responses were not deleted from the sample but were recoded as missing. For purposes of analytic clarity, the perceived climate change health risk variable was converted into a dichotomous format. This decision was based on both methodological and conceptual considerations. Additionally, the distribution of responses was notably skewed, with limited variation across certain response categories, making the assumption of linearity problematic for regression models.

For demographic variables considered as covariates, age, sex, education, income, and marital status were used. Confounding variables include demographic factors such as gender as “On your original birth certificate, were you listed as male or female?”, Sexual identity as “Do you see yourself as…?”, employment status as “Which of the following best describe your current occupational status?”, Marital status as “What is your marital status?”, educational attainment as “What is the highest grade or level of schooling you completed?”, income as “Thinking about members of your family living in this household, what is your combined annual income, meaning the total pre-tax income from all sources earned in the past year?” Education-level data was categorized into four groups: “<Post High School” versus “Some College” versus “College Graduate” versus “Postgraduate”. Sexuality was categorized as “Heterosexual” versus “Not Heterosexual”. Marital Status was categorized as “Married” versus “Not Married” versus “Others”. Income information was computed by the poverty income ratio (PIR), which is also an indicator of income relative to the economic needs of a household. This is a polytomous variable that was achieved by calculating annual fluctuations in household size and cost of living and monitoring the consumer price index concerning household income and federally established poverty limitations. PIR levels were defined as low income (PIR < 1), middle income (1 ≤ PIR < 4), and high income (PIR ≥ 4) and dichotomized for analysis with a cutoff point of 1. These variables are critical for understanding how social and economic factors may influence individuals’ concerns about climate change and its mental health impacts. Notably, by considering these confounding variables, the study aims to control for factors that could influence the relationships between climate change awareness and mental health outcomes. Although the HINTS-6 dataset includes racial data, this variable was excluded from our analysis due to a lack of consistent associations reported in prior literature and the absence of statistically significant relationships observed in our preliminary analyses.

### 2.6. Data Analysis

For the data analysis, we focused on analyzing secondary data from the HINTS-6 to explore relationships between perceived climate change health risk and mental health outcomes. The study was based on a cross-sectional design, which is appropriate for examining correlations and associations at a specific point in time. Based on data manipulation, no response was deleted to maintain the statistical rigor. Given the vast amount of data available in the HINTS-6 dataset, we aimed to extract meaningful insights regarding perception of climate change and its connection with mental health, particularly anxiety and depression. This quantitative approach involved the use of various statistical techniques to test the study’s hypotheses and interpret the data within the context of the survey’s scope. The first step in the analysis involved descriptive statistics to summarize the sample characteristics. This included measures such as frequencies, means, and standard deviations for the demographic variables (e.g., age, gender, income, education) and the primary study variables (e.g., awareness of climate change, depression/anxiety diagnoses, and worry). These descriptive analyses provided an overview of the sample’s composition and the distribution of key variables, helping to identify patterns that may guide further statistical tests. Additionally, the use of visualizations such as histograms and bar charts will aid in understanding the data’s distribution, while also highlighting any potential outliers or anomalies. To examine the relationships between the dependent and independent variables, We employed inferential statistical techniques, beginning with chi-square tests and independent samples *t* tests. The chi-square test was used to analyze categorical variables, such as the relationship between mental health diagnoses (e.g., depression, anxiety) and sociodemographic factors like gender. This helped identify any significant associations between categorical variables. For more complex relationships, complex samples logistic regression will be the primary statistical method used to assess the influence of multiple independent variables on binary outcomes such as whether a respondent has been diagnosed with depression or anxiety. This technique is particularly useful because it can handle multiple predictors simultaneously, including demographic factors (e.g., gender, income, education) and perception of climate change. Additionally, the technique is ideal because the probability of being selected for the sample is not equal for all participants. Logistic regression allowed for an estimation of odds ratios, which indicated the likelihood of a mental health diagnosis given different levels of climate change concern, while controlling for potential confounding variables. This analysis provided a clearer understanding of how perceived climate change health risk may be connected to mental health outcomes, accounting for other sociodemographic factors that could serve as confounders for the relationships. These include factors such as gender, race, marital status, and income, which could affect both mental health and perceived climate change health risk. By controlling for these variables in the regression models, the analysis provided a more accurate estimate of the relationship between perceived climate change health risk and mental health outcomes. Additionally, sensitivity analyses were conducted to check for the stability of the results across different subgroups (e.g., male vs. female). The ultimate goal of this analysis was to offer actionable insights into how climate change may impact public health, particularly among vulnerable populations.

### 2.7. Ethical Consideration

Ethical procedure is important for public health research. This study received institutional review board approval from Walden University with approval number 01-21-25-1188599.

## 3. Results

### 3.1. Demographic Attributes Overview

Table 1 presents the demographic characteristics of the HINT-6 study population. The average age of participants was 55.6 years, with a standard deviation of 17.4 years. Gender distribution showed that 39.52% were male and 60.48% were female. Employment status indicated that 50.86% were employed, while 49.14% were unemployed. Regarding marital status, 51.49% were married, 29.28% were not married, and 19.24% identified as divorced, widowed, or separated. Educational attainment varied, with 32.11% having less than a high school education, 21.28% some college experience, 27.67% holding a college degree, and 18.94% having postgraduate education. In terms of sexual orientation, 92.22% identified as heterosexual. Annual income distribution revealed that 43.66% earned less than $50,000, 29.66% earned between $50,000 and $99,999, and 26.68% earned more than $100,000. Notably, 26.40% of participants reported experiencing depression or anxiety, while 78.28% indicated a climate change perception. See Table 2 for the sociodemographic factors distribution based on the disclosure of depression or anxiety.

### 3.2. Odds Ratio Estimates for Sociodemographic Attributes and Mental Health

Table 3 details the both crude and adjusted odds ratio estimates for the association between sociodemographic attributes, climate change awareness, and the likelihood of experiencing depression or anxiety. For context of the crude odds ratio, see Table 3 above. In terms of adjusted odds ratios, the analysis indicates that age has an adjusted odds ratio of 0.973 (CI: 0.968–0.978), suggesting a continued decrease in the odds of reported mental health issues with age. For gender, females compared to males had an adjusted odds ratio of 0.586 (CI: 0.505–0.679), indicating a reduced likelihood of reporting depression or anxiety when controlling for other factors. Being employed compared to unemployment resulted in an adjusted odds ratio of 0.598 (0.507–0.704), suggesting that unemployment significantly increases the odds of mental health challenges. For marital status, being single compared to being married resulted in an adjusted odds ratio of 1.582 (1.320–1.895), while not being divorced compared to being married showed an adjusted odds ratio of 1.111 (0.917–1.345). Educational comparisons revealed that those with less than post high school exposure versus postgraduate education had an adjusted odds ratio of 1.007 (CI: 0.810–1.253), while those with some college experience compared to postgraduate showed an adjusted odds ratio of 1.068 (CI: 0.853–1.336). For college graduates compared to postgraduate individuals, the adjusted odds ratio was 0.993 (CI: 0.812–1.214). For sexual orientation, non-heterosexual individuals had an adjusted odds ratio of 2.691 (CI: 2.125–3.407) compared to heterosexual individuals, indicating a strong association with mental health issues. Income analysis revealed that those earning less than $50,000 compared to more than $100,000 had an adjusted odds ratio of 1.169 (CI: 0.951–1.437), while earning between $50,000 and $100,000 compared to more than $100,000 had an adjusted odds ratio of 0.884 (CI: 0.729–1.072). Finally, individuals with awareness about climate change compared to those without awareness had an adjusted odds ratio of 1.392 (1.160–1.671), further emphasizing the importance of Climate change awareness in mental health outcomes. Tests for interaction effects revealed that this relationship did not significantly vary across key sociodemographic attributes such as age, gender, income, and education. This suggests that the association between climate change awareness and mental health outcomes is relatively uniform across different population subgroups.

## 4. Discussion

In recent years, the spotlight has been on the relationship between climate change awareness and depression, anxiety, and associated mental health issues. We conducted comprehensive investigation into the relationships between a variety of sociodemographics, climate change awareness, and a reported response of previous depression or anxiety. While previous research has established some correlation between these variables and health outcomes, its nuances regarding sociodemographic factors of interest are reported here, with significant results pertaining to age, gender, employment status, marital status, education, income, and sexual orientation. Moreover, awareness of climate change itself appears to drive mental health, which may imply that greater climate change awareness may reduce climate change-related anxiety and depression [10]. Thus, the context of sociodemographic attribute portrays the importance of this study findings.

The sociodemographic profile of the HINT-6 study population appears to capture important trends relevant to mental health outcomes. Participants had a mean age of 55.6 years, which is a demographic that may have unique mental health needs or challenges [35] and may report on climate anxiety [15,36]. The crude odd ratio of age showed that the likelihood of depression or anxiety was lower in older individuals, with an odds ratio of 0.981. This indicates that older individuals report mental health issues marginally less likely than younger individuals and correlates with prior literature suggesting resilience and adaptive mechanisms improve with age [37,38]. However, this association between age and mental health could be dependent on several psychosocial factors, including life experience and social support networks. Similarly, the adjusted estimates for age (AOR= 0.973) further supports the idea that older individuals remain less vulnerable to mental health distress.

There was gender disparate distribution in terms of the study sample with 60.48% being female, which is consistent with the literature, where women are found more vulnerable to mental health threats elicited by climate change [39,40,41]. Such susceptibility could arise from social constructs and gender roles around emotional expression and coping strategies. For example, men may use avoidance techniques, which can result in various forms of depression, whereas women frequently turn to social support during stressful times [15]. This research emphasizes how important it is to modify mental health resources to accommodate the distinct requirements of various gender groups. Based on the analysis, females have higher crude odds ratios for mental health issues related to Climate change awareness (OR = 1.925), suggesting that an almost two-fold higher number of females reported symptoms of depression or anxiety compared to males. This gendered trend in mental well-being is strongly supported in the literature, with women manifesting higher rates of depression and general anxiety consistently than men, which may even be associated biologically with hormonal changes and increasing expectations of women for positive child/parental and social roles [42,43]. Interestingly, females had a statistically significant lower odds of depression or anxiety (AOR = 0.586) after adjusting for other sociodemographic factors. This suggests that the gender gap noted in the mental health outcomes was protective when other sociodemographic factors were considered; thus, suggesting the possible influence of additional risk factors like marital status or employment. These findings emphasize the crucial nature of offering resources for mental health that are tailored towards gender.

Employment status remains another crucial factor that correlated with mental health outcomes. We noted that 50.86% of participants were employed and that the odds of reporting depression or anxiety were inversely associated with employment status. Unemployment has been suggested to be associated with increased financial hardship, decreased social contacts, and decreased feelings of purpose in life, which are all contributing factors to mental health disorders [44]. Employment status was another factor closely linked with mental health. For those without jobs, the crude odds ratio was OR = 0.829, suggesting that employment may have some protective effects against anxiety or depression. Based on this analysis, the odds of depression or anxiety for unemployed persons were significantly greater than those with full-time employment, consistent with recent work reporting that unemployment is related to increased incidence of mental health challenges [45]. But when controlling for other sociodemographic factors, being unemployed was associated with particularly high odds of having mental health problems (AOR = 1.673). This finding is consistent with earlier studies showing a link between social positions, job security, and better mental health outcomes [45,46]. Furthermore, there is a negative correlation between employment and awareness of climate change, suggesting that those who are working may have stronger coping strategies, resources, and support networks in place to handle the different stresses that climate change may bring [47,48]. This insight highlights how mental health in the context of climate change cannot be viewed outside socioeconomic factors.

Findings concerning marital status also merit attention as it turned out to be the more important factor in mental health outcomes. Single or unmarried individuals had higher odds of suffering from depression or anxiety than those who were married, indicating a statistically higher chance of getting depression or anxiety for married individuals. This finding dovetails with research showing that strong social support systems, prevalent in marital relationships, are protective factors for mental health [49,50]. In contrast, divorced or separated individuals relative to married individuals had a nonsignificant trend, but tended towards being at higher odds of experiencing a mental health issue. This pattern did not align with previous research finding that showed protective effect of marriage on mental health [51]. Thus, unmarried or separated people may not have this emotional support, which could contribute to increased anxiety or depression, and a partner’s emotional support can buffer stress and promote resilience to environmental stressors, like climate-related fears. This is important, as social connections have been shown to buffer against the mental health impacts of climate change, and isolation may worsen feelings of hopelessness and despair [52].

Education was another variable strongly related to mental health based on this study. Although higher education levels usually correlate with more favorable mental health outcomes, the data showed mixed results. Those with less than a high school diploma reported rates of depression or anxiety similar to participants with postgraduate degrees. The crude odds ratio for education level (less than high school vs. postgraduate education) was not statistically significant (OR = 1.189). However, the adjusted analyses depicted that less than high school education was associated with reported depression or anxiety (AOR = 1.314), when comparing individuals with postgraduate education. Thus, raises important questions about how education is related to climate change awareness in the first place and how that, in turn, affects mental health. This lends some support to the hypothesis that education leads to enhanced cognitive and emotional resources that can assist individuals to cope with stressors, including challenges to mental health [53]. On the other hand, evidence in the literature also suggests that education is associated with perception of climate change in the sense that a more educated population is typically more aware of the impacts of climate change, and thus may face higher levels of anxiety [47,48]. Hence, this suggests that a higher level of education, though very helpful to understand climate-related issues, can also contribute to greater worries associated with future uncertainties. Therefore, despite positive action through educational initiatives, there should be accompanying mental health support, as climate change brings about psychological consequences as a result.

The role of sexual orientation in the public health and population health hemisphere has been well documented in contemporary times [54]. This study showed that non-heterosexual individuals reported substantially higher odds of depression or anxiety than heterosexual individuals (OR = 3.335), therefore suggesting that they were approximately three times more likely to have a depression or anxiety diagnosis compared to heterosexual individuals. This finding was corroborated after adjustment of confounding variables (AOR = 2.691), highlighting the increased burden of poor mental health amongst non-heterosexual people [55,56]. Evidence in the literature suggests that sexual minorities have a higher rate of mental illness than heterosexuals due to factors including discrimination, stigma, and minority stress [56].Such a difference is expected for communities that are already marginalized as LGBTQ+ individuals who have unique stressors they regularly face, like discrimination and stigma from society, which contribute to their mental health issues [57,58]. These climate change-related health risks, including climate change anxiety, warrant attention to mental health for sexual minority populations engaged in climate action. This fact highlights the need for mental health interventions that are LGBTQ+-inclusive and trauma-informed in the face of climate change.

Economic distress is a well-established risk factor for mental health, with financial insecurity causing feelings of hopelessness and depression [59,60]. In the context of climate change awareness, the association between income and mental health outcomes also needs to be considered. We found that people with climate change awareness making less than $50,000 were much more likely to be depressed or anxious compared with individuals earning more than $100,000 (OR = 1.517). Following considerations for other sociodemographic attributes and outcomes, the findings remained same (AOR = 1.169). These results are consistent with a substantial literature showing that socioeconomic status is a robust predictor of mental health, with at-risk groups being more likely to report psychological distress [59,60]. Furthermore, those with lower incomes may not have access to social support networks, health care, education, and other resources that can lessen the effects of climate change irrespective of their awareness to this challenge [61,62]. These situations capture the intersection of mental health, socioeconomic position, awareness and climate change, necessitating comprehensive approaches that take interconnected issues into consideration.

The association between climate change awareness and mental health outcomes, particularly depression or anxiety, is an emerging area of concern, especially as environmental events intensify. We noted that those who possessed awareness of climate change were more likely to be depressed or anxious, indicating that ignorance of climate change may protect against mental health concerns triggered by environmental stressors [63]. This relationship remained in adjusted models controlling for confounding variables. This supports the notion that awareness, while critical for fostering engagement and adaptive behavior, may also increase psychological vulnerability, possibly due to heightened concern, perceived helplessness, or eco-anxiety related to climate threats. Importantly, there was no significant interaction between this relationship and sociodemographic attributes such as age, gender, income, or education, indicating that the association between climate change awareness and mental health outcomes appears consistent across diverse population subgroups. This underscores the need for public health efforts that not only raise awareness but also provide supportive messaging and coping strategies to buffer psychological distress. These outcomes underscore the failure of public health education in addressing the psychosocial consequences of climate change [64]. This is consistent with the literature showing that climate change education and awareness may not be associated with lower rates of climate-related anxiety [65]. Contrary to this, Elzohairy et al. (2024) found that a psychoeducational program about climate change led participants to estimate higher health-related risks from climate change, but paradoxically reported lower levels of climate-related emotional distress [66]. This suggests that increased knowledge may enhance cognitive risk appraisal while providing a sense of control or coping that reduces emotional burden. However, the study’s generalizability is limited by its small sample size, focus on older adults, and relatively low educational attainment among participants. Alternatively, there is literature suggesting that increased climate change awareness may prompt a range of adaptive behaviors that promote a sense of agency and reduce feelings of helplessness [67]. Though further studies is still needed, the findings suggest that a greater understanding of climate change can empower people and strengthen their urgency and involvement in pro-environmental behavior [68]. This empowerment can help alleviate helplessness, which frequently comes along with inducing anxiety about environmental adverse outcome. However, it also needs to be understood that greater awareness can translate to increased anxiety if climate change is perceived as an inevitable threat [22,27,69]. Therefore, while climate change education is critical, it should be integrated with resilience- and coping-building strategies.

## 5. Strength & Limitations

The use of HINTS-6 dataset for this study was a major strength of this study as it included a large, national sample, allowing an understanding of awareness and depression or anxiety among the general population regarding climate change. Hence, this created a strong basis for identifying such relationships. Another strength stems from my providing valuable insights that can inform policy decisions and interventions aimed at enhancing climate change literacy and action. However, the study has several limitations. First, as a cross-sectional analysis, it can identify correlations but cannot establish causality whether increased awareness of climate change directly causes depression or anxiety, or whether pre-existing mental health conditions influence people’s awareness of climate change. Moreover, the HINTS-6 dataset relies on self-reported data, which may be prone to reporting bias, especially when it comes to mental health conditions like depression and anxiety, which individuals may underreport due to stigma or lack of awareness. This study is also limited by the use of single-item measures for key variables, which may oversimplify complex constructs such as perceived health impacts of climate change and mental health status. The question assessing perceived harm from climate change may be interpreted in various ways and lacks nuance in distinguishing between awareness, knowledge, and personal concern. Additionally, the mental health outcome is based on lifetime clinical diagnosis, which doesn’t take cognizance of current symptom status, nor severity of the problem. The potential for underdiagnosis in some populations also limits the accuracy and generalizability of the findings. Another limitation is that the dataset might not fully represent the breadth of psychological responses to climate change, making it difficult to gauge the degree of both awareness of the issue and real symptoms of depression or anxiety, such as eco-anxiety or other more specific forms of distress. Finally, while the sample is nationally representative, it may not account for regional variations in climate impacts or mental health care access, potentially masking significant localized effects.

## 6. Implications for Interventions in Mental Health

These results have important implications for the provision of mental health interventions given the context of climate change. Since demographic factors affect mental health outcomes differently, tailored interventions that consider individual circumstances are needed. Additionally, programs designed to assist unemployed individuals, for example, can knit mental health resources into their interventional plans to mitigate the unique mental health stressors that accompany unemployment, especially in harrowing socioeconomic conditions spurred by climate change. In the same way, mental health programs for non-heterosexual individuals need to consider the extra stressors they face in the context of climate change, and the implications of awareness status. Additionally, climate change awareness educational methods must incorporate mental health as well in their prevention designs. These programs may help buffer against anxiety and depression that might stem from increased awareness of climate problems, through teaching coping strategies and resilience. Thus, one relatively new area of interest linking climate change and human well-being is climate change awareness and mental health (e.g., depression, anxiety) such as the psychological risk associated with climate change as predictors of mental health status. These results show a clear link between climate change awareness and mental health, with broad implications for research, education, public health practice, and policy. We will comment on some of the social implications of these findings in these three areas.

### 6.1. Research

Studying the link between climate change awareness and mental health can help better understand the mental health impacts of climate change. The researchers can lead to more nuanced insights by generating inferences through a smooth relationship between awareness and mental health status and then meaningful findings for further studies about demographics and risk factors. For example, it is well-documented that individuals of low to lower to middle socioeconomic status and those with limited access to mental health resources are disproportionately impacted by climate anxiety [36,70]. A key to recognizing when someone is at risk is understanding these dynamics to identify vulnerable populations and create tailored interventions. Additionally, it can inform predictive models that determine mental health risks caused by climate change through continued research. Bridging these disciplines can allow researchers to return to the rapid pace of climate change by using frameworks to inform public health initiatives about interventions that can be implemented to reduce the mental health effects [71]. Research is well explained and provides data useful to practitioners and policymakers.

### 6.2. Education

The results about the mental health effects of climate change awareness may be valuable for educational efforts across all levels. Schools and universities can develop curricula on climate literacy—again, with a focus on the psychological effects of climate change. The populace can be better equipped to face obstacles in the future by learning about the effects of climate change and coping mechanisms for anxiety and sadness. Furthermore, initiatives for community education can also raise awareness among many groups, particularly those who are most affected by climate change. Thus, whilst this article focuses specifically on climate change, the broader applicability of the suggested intervention is useful: giving workshops and resources on climate change together with its impact on mental health can foster resilience and coping mechanisms, promoting well-being in communities facing environmental problems [72]. When people become more aware of these issues they may take pro-environmental actions, too, which can improve mental health by instilling a sense of agency and connectedness to a community.

### 6.3. Public Health Practice

While these challenges require critical solutions, we can no longer afford a modular approach to climate change adaptation. The research can help health practitioners advocate for mental health resources that meet the needs of communities experiencing climate-related stressors. Evidence in the literature described integrating mental health screenings and support services into such climate resilience programs as one way climate adaptation programs can help address and mitigate the individual psychological burden of climate events. Additionally, community leaders, environmental scientists, and mental health professionals can work together to develop all-encompassing programs that address the psychological and physical effects of climate change. This enables more successful solutions and provides a more thorough grasp of the problems communities are facing.

### 6.4. Policy Implications

It may also inform policies around mental health and climate change. Advocacy for climate and health policies can demonstrate how the association between climate change awareness and mental health outcomes can be used by policymakers to ensure policy is driven by evidence. There are several opportunities to incorporate mental health into climate action, including initiatives to incorporate psychological well-being considerations into climate action plans [73]. These findings can also inform funding allocations for mental health services in the context of climate change. Additionally, this study highlights the importance of factors such as employment and income, as such fair wages carry significant policy implications. It emphasizes that individuals facing socioeconomic hardships are particularly vulnerable, and policies should consider prioritizing this group for enhanced mental health support. This observation aligns with the data, which indicate that these individuals are among the most at risk. Thus, by acknowledging the mental health impact of climate change, policymakers can help ensure resources are allocated for mental health initiatives, especially in vulnerable communities that are in many ways already bearing the brunt of environmental turnover. It is a sound investment that ultimately can drive better mental health outcomes and community resilience.

## 7. Conclusions

Climate change awareness and mental health (including depression and anxiety) are closely related; at the same time, this relationship is multifactorial and not yet fully elucidated. Hence, the need to consider sociodemographic factors like gender, employment status, marital status, education, sexual orientation, and income levels when examining mental health outcomes based on climate change awareness. As the effects of climate change continue to unfold, it is crucial for mental health experts and policymakers to join hands in devising targeted strategies that cater to the specific needs of various communities. Thus, climate change awareness should be promoted in a parallel path as well as improving mental health services to boost the capacity of individuals to deal with the challenges posed by climate change through promoting supportive psychological resilience. Nonetheless, further studies are indeed required corroborate this study findings, and to understand the nature of the association.

## Figures and Tables

**Table 1:** Attributes of the HINTS-6 Population.

Characteristics	Sample (N = 6154)	
	*N*	%
Age (Mean ± SD)		55.60 ± 17.43
Gender		
Male	2293	39.52
Female	3509	60.48
Employment		
Yes	2952	50.86
No	2852	49.14
Marital status		
Married	2979	51.49
Not married	1694	29.28
Divorced/widowed/separated	1113	19.24
Education		
<Post high school	1860	32.11
Some college	1233	21.28
College graduate	1603	27.67
Postgraduate	1097	18.94
Sexual orientation		
Heterosexual	5212	92.22
Not heterosexual	440	7.78
Annual income		
<$49,999	2396	43.66
$50,000–$99,999	1628	29.66
>$100,000	1464	26.68
Depression or anxiety		
Yes	1565	26.40
No	4364	73.60
Climate change awareness		
Yes	3924	78.28
No	1089	21.72

**Table 2:** Sociodemographic attributes of HINTS-6 based on Depression or Anxiety.

Characteristics	Depression or Anxiety		
	Yes	No	*p*-value
	N (%)	N (%)	
Gender			<0.0001
Male	436 (28.57)	1842 (43.50)	
Female	1090 (71.43)	2392 (56.50)	
Employment			0.0137
Yes	736 (48.20)	2196 (51.88)	
No	791 (51.80)	2037 (48.12)	
Marital status			<0.0001
Married	679 (44.55)	2287 (41.19)	
Not married	483 (31.69)	1188 (28.15)	
Divorced/widowed/separated	362 (23.75)	745 (17.65)	
Education			0.4085
<Post high school	490 (32.17)	1353 (32.02)	
Some college	344 (22.59)	880 (20.82)	
College graduate	416 (27.31)	1177 (27.85)	
Postgraduate	273 (17.93)	816 (19.31)	
Sexual orientation			<0.0001
Heterosexual	1284 (85.71)	3896 (94.63)	
Not heterosexual	214 (14.29)	221 (5.37)	
Annual income			<0.0001
<$49,999	732 (49.66)	1643 (41.31)	
$50,000–$99,999	393 (26.66)	1225 (30.80)	
>$100,000	349 (23.68)	1109 (27.89)	
Climate change awareness			<0.0001
Yes	1130 (84.71)	2766 (75.91)	
No	204 (15.29)	878 (24.09)	

**Table 3:** Univariate and Multivariate Analysis for Climate Change Awareness, Covariates and Depression or Anxiety.

Dependent Variable	Odds Ratio	
	Crude (95% CI, *p*)	Adjusted (95% CI)
	Yes vs. no	Yes vs. no
Age	0.981 (0.978–0.984, <0.0001)	0.973 (0.968–0.978, <0.0001)
Gender		
Male	Reference	Reference
Female	1.925 (1.696–2.185, <0.0001)	0.586 (0.505–0.679, <0.0001)
Employment		
Yes	1.159 (1.031–1.303, 0.0137)	0.598 (0.507–0.704, <0.0001)
No	Reference	Reference
Marital status		
Married	Reference	Reference
Not married	1.369 (1.195–1.569, <0.0001)	1.582 (1.320–1.895, <0.0001)
Divorced/widowed/separated	1.637 (1.406–1.905, <.0001)	1.111 (0.917–1.345, 0.1914)
Education		
<Post high school	1.189 (0.985–1.436, 0.2427)	1.007 (0.810–1.253, 0.8799)
Some college	1.216 (0.992–1.490, 0.1585)	1.068 (0.853–1.336, 0.4520)
College graduate	1.925 (0.885–1.294, 0.4539)	0.993 (0.812–1.214, 0.6930)
Postgraduate	Reference	Reference
Sexual orientation		
Heterosexual	Reference	Reference
Not heterosexual	3.335 (2.676–4.155, <0.0001)	2.691 (2.125–3.407, <0.0001)
Annual income		
<$49,999	1.517 (1.293–1.781, <0.0001)	1.169 (0.951–1.437, 0.0765)
$50,000–$99,999	0.983 (0.823–1.174, 0.0828)	0.884 (0.729–1.072, 0.0780)
>$100,000	Reference	Reference
Climate change awareness		
Yes	1.758 (1.487–2.079, <0.0001)	1.392 (1.160–1.671, 0.0004)
No	Reference	Reference

## Data Availability

The data presented in this study are available at this link—https://hints.cancer.gov/data/download-data.aspx (accessed on 25 January 2025).
